# An assessment of variables affecting transition readiness in pediatric rheumatology patients

**DOI:** 10.1186/s12969-015-0040-x

**Published:** 2015-10-13

**Authors:** Catherine April Bingham, Lisabeth Scalzi, Brandt Groh, Susan Boehmer, Sharon Banks

**Affiliations:** Department of Pediatrics, Division of Pediatric Rheumatology, Penn State Hershey Children’s Hospital and Penn State College of Medicine, 500 University Drive, HS83 Hershey, PA USA; Department of Public Health Sciences, Division of Biostatistics and Bioinformatics, Penn State University, Hershey, PA USA; Department of Internal Medicine, Division of Rheumatology, Penn State Hershey Medical Center and Penn State College of Medicine, Hershey, PA USA

**Keywords:** Self-care assessment, Adolescent independence, Health care transition, Transition readiness

## Abstract

**Background:**

We sought to identify which adolescent patient characteristics might lead to subjective reported independence in accessing medical care when patients transition from pediatric to adult medicine.

**Methods:**

Pediatric and adult rheumatologists were asked which pediatric patient characteristics they believed would improve transition to adult medical care. Based on these responses, a questionnaire was created and administered to 76 teenage/young adult patients in a pediatric rheumatology clinic. The first set of questions included demographic, disease features, and life skills questions. The second set of questions pertained to self-reported independence in managing medical care. Data was analyzed to see if there were any significant associations between an individual’s response to demographic, disease feature, or life skills questions and the independence outcome questions.

**Results:**

In our study, older age correlated with self-reported independence in almost all questions asked regarding accessing medical care. Other patient characteristics that were associated with increased self-perceived autonomy included having a younger parent, having a family member with a similar disease, longer disease duration, having a comorbid non-rheumatic diagnosis, and having had a summer job.

**Conclusions:**

The patient characteristics that we found associated with self-reported independence in obtaining medical care should be considered when determining which patients might be more likely to make a successful transition.

## Background

Health care transition is described as the “purposeful, planned movement of adolescents and young adults with chronic physical and medical conditions from child centered to adult oriented health care systems” [1]. However, this type of “planned movement” is not easy. Many children and young adults with rheumatic disease have persistent active disease as adults and need ongoing adult rheumatology subspecialist care. Up to 60 % of patients with juvenile idiopathic arthritis, the most common childhood rheumatic disease, are not in remission at long-term follow up [2]. In fact, studies of adult outcomes in other childhood-onset rheumatic diseases also show high rates of active disease [3]. Sadly, studies have demonstrated lack of successful health care transition of pediatric rheumatology patients [4, 5]. When they are transitioned, young people often are lost to follow up or demonstrate poor adherence to treatment. Since adverse outcomes are possible if transition is not accomplished properly, quality improvement efforts should focus on achieving successful transition of care.

An American Academy of Pediatrics (AAP) survey found the following barriers to transition of care cited by general pediatricians: lack of knowledge of community resources, fragmentation of adult health care, lack of young adults’ knowledge about their own condition and skills to self-advocate during healthcare visits, lack of primary and specialty providers, close bond between health care provider and family, lack of office skills in transition, and lack of reimbursement for transition activities [6]. Other barriers noted include lack of care coordination infrastructure and lack of electronic medical record functionality for transition elements [7]. Adult providers may not feel comfortable caring for patients with pediatric-specific diagnoses [8]. Expectations and care delivery may differ between pediatric and adult providers. Furthermore, unique aspects of adolescent development may not be taken into account. Pediatric providers are often family-oriented and willing to coordinate and supervise a treatment plan they devise and initiate [9]. Adult providers, on the other hand, are often individual focused (preferring to interact with the patient rather than family), more accepting of patient refusal of treatment, and may provide less supervision of adherence to treatment [9].

In addition to different pediatric and adult clinician styles, transition into adulthood for young people with rheumatic disease is also difficult due to parental and patient psychosocial factors. Parents may overprotect and provide excessive supervision of care. Young people with chronic disease may have poor development of peer relationships, a lack of counseling on health risk behaviors such as drinking alcohol while on medications, rebellious behavior, denial of the need for care/treatment, and poor vocational readiness [10, 11]. A formal transition program might help overcome some of these difficulties. For example, one program for transitional care established for patients with JIA showed improvement in Health Related Quality of Life [12]. This transitional program also resulted in improved adolescent and parent knowledge, satisfaction, and pre-vocational readiness markers [12]. A recent review of existing transition programs for youth with chronic diseases demonstrated that joint pediatric /adult clinics, specific young adult clinics, and patient education programs were the key components leading to better outcomes for young people with diabetes [13]. Knowing that similar transition difficulties exist regardless of what chronic disease is being treated [14], we sought to identify which background factors might lead to increased adolescent self-reported autonomy in accessing medical care. We hypothesized that youth with some autonomy in life skills, such as having a summer job, might be more likely to be independent in obtaining medical care.

## Methods

Pediatric and adult rheumatologists at Penn State Hershey Medical Center were asked by survey which pediatric patient characteristics they believed were required to ease transition to adult medical care. Two pediatric rheumatologists, three adult rheumatologists, and two combined medicine and pediatric trained adult/pediatric rheumatologists all from one academic medical center completed our survey. These physicians had been practicing rheumatology from 4 to 20 years, and all routinely take care of young adults who are transitioning or have transitioned. Due to a lack of available published validated transition readiness questionnaires at the time this study was conceptualized, in 2007, we created a questionnaire using the results from our survey.

The 41 item questionnaire was comprised of two types of questions. The first set of questions included demographic, disease features, and life skills questions. Demographic questions included age, gender, race, age of parent, if adolescent was living at home, and family income. Disease specific information was collected including diagnosis (more than one was allowed, e.g. secondary juvenile fibromyalgia), medications prescribed, whether lab monitoring was required, and disease duration. Patient pain score and global assessment of overall well-being were included. Life skill questions asked whether the patient had a driver’s license, what grades he/she attained in school, and if the patient ever had a summer job. The second set of questions pertained to independence in managing medical care. Examples of such questions included the following: “Do you take your medications as prescribed without someone reminding you?”; “When you don’t feel well, who usually phones the doctor?”; “Who would you call first if you ran out of medication and had no refills?”; and “Who makes your routine doctor’s appointments?”. Institutional Review Board approval and informed consent was obtained from all participants. Teenage/young adult rheumatology patients with any rheumatic condition were invited to participate in the study at the end of a pediatric rheumatology clinic visit from 2008 until 2010. These patients filled out the questionnaire independently, without parental input, while in the exam room.

Data was analyzed to see if there were any significant associations between an individual’s response to demographic, disease feature, or life skills questions and the independence outcome questions. Categorical and continuous variables were analyzed using Pearson’s Chi-square and two-sample t-tests, respectively. In circumstances when a significant variable had the potential to be related to age, e.g. having a summer job or a driver’s license, disease duration, or parents’ age, logistic regression analyses were also performed to control for the patient age and to evaluate for this potential interaction. When examining the effect of having a driver’s license on the independence outcome measures, only those subjects who were 16 years of age or greater were included in the analysis.

The primary outcome was a measure of independence – taking medications without being reminded. With a null hypothesis of 0.3 and a type I error rate of 0.05, the calculated power was 99 %. Statistical software package, SAS version 9.3 (copyright by SAS Institute, Cary, NC) and JMP (JMP 10. SAS Institute Inc. Cary, NC) was used to analyze the data. Descriptive statistics were generated including means and standard deviations for continuous variables and frequency tables for the categorical variables. Differences between groups were characterized using contingency table analysis. *P* values ≤0.05 were considered significant.

## Results

A total of 76 patients were surveyed. Demographics are shown in Table 1. The mean age of our cohort was 17 ± 1.8 years (range = 12–21 years; only one patient was in the 12–13 year old range). Twenty-six percent of patients were 14–15 years old. Fifty-one percent of our study population was 18 years of age or older. Our population was largely female (71 %), Caucasian (94 %), and non-Hispanic (90 %). Eighty-six percent of patients had a parent older than 40 years of age. Sixty patients (80 %) had JIA, 6 (8 %) had lupus, and 12 (16 %) had fibromyalgia. Twenty-eight percent of patients had other rheumatic diagnosis including ANCA-associated vasculitis, Behcets, post-streptococcal reactive arthritis, Rheumatic fever, joint hypermobility syndrome, idiopathic uveitis, scleroderma, chronic fatigue, dysautonomia, and reflex sympathic dystrophy. Mean disease duration was 4.5 ± 3.3 years (range = <1 to 15 years). Fifty-four percent of patients had a family member with a “similar” disease. Most patients reported having a good understanding of their disease and of their medicines, 94 % and 85 %, respectively. Ninety-one percent of patients were still in school, and all but one patient still lived at home. Of patients in school, 82 percent were in middle or high school, and 18 percent were in college. Fifty-seven percent had had a summer job, and 43 % had a driver’s license.

**Table 1:** Demographic characteristics

Characteristic (*N* = 76)	Value
Demographics	
Age (years) (mean ± s.d.)	17.0 ± 1.8 years
Gender	
Female	71 % (54)
Male	29 % (22)
Race	
Caucasian	94 % (67)
African-American	4 % (3)
Pacific Islander	2 % (1)
Ethnicity	
Hispanic	10 % (7)
Non-Hispanic	90 % (29)
Parent > age 40	86 % (60)
Guardian education	
High school or higher	86 % (60)
College and higher	31 % (22)
Number of siblings (mean ± s.d.)	2.2 ± 1.3
Family member with a similar disease	54 % (39)
Disease Associated Variables	
Disease duration (years) (mean ± s.d.)	4.5 ± 3.3
Diagnosis	
Juvenile idiopathic arthritis	80 % (60)
Systemic lupus erythematosus	8 % (6)
Juvenile fibromyalgia (primary or secondary)	16 % (12)
Other	28 % (21)
Taking medications	84 % (64)
Routine labs required	58 % (42)
Good self-reported understanding of disease	94 % (68)
Good self-reported understanding of medications	85 % (62)
Patient Education/ Life Skills and Behaviors	
Currently living with guardians	99 % (75)
Currently attending school	91 % (68)
Grade level currently attending	
8^th^-10^th^ grade	35 % (24)
11-12^th^ grade	47 % (32)
College	18 % (12)
Summer Job (ever)	57 % (43)
Have a driver’s license	43 % (33)
Tobacco Use (yes)	4 % (3)
Independence Outcomes	
Patient indicated he/she would call the doctor him/herself if not feeling well.	10 % (7)
Patient reported he/she would call the doctor or pharmacy first if had no refills on medication.	57 % (41)
Patient makes own routine doctor’s appointments.	18 % (13)
Patient calls him/herself to cancel a doctor’s appointment if cannot make it.	70 % (51)
Patient reports he/she takes medications as prescribed without someone reminding him/her.	87 % (62)

In regards to the independence outcomes, eighty-seven percent of patients reported taking their medicines without being reminded. Seventy percent reported that they would call the physician’s office themselves to cancel if they were unable to attend a scheduled visit, but only 18 percent made routine doctor appointments by themselves. Fifty-seven percent stated that they would independently call the physician’s office or the pharmacy if they required a refill, instead of having a family member do this. Only 10 % (7 respondents) reported that they would be the party responsible for calling the physician’s office if they were not feeling well. We found that certain demographics, disease features, and life skills were significant for young adults with increased self-reported independence in obtaining medical care (see Table 2). There were no significant interactions with age and significant variables, but certain variables were found to no longer be significant when controlled for age (see Table 2). Table 3 summarizes the patient variables associated with greater self-reported independence in accessing the health system.

**Table 2:** Factors associated with increased self-reported autonomy in medical care

Independence outcome				
		Independent action	Dependent action	*p*-value
	Variable			
When you don’t feel well, who usually phones the doctor? (“me” was considered independent action)	Age (years)	18.80 ± 1.32	16.70 ± 1.63	0
	Summer Job			
	Yes	100 %	51.50 %	0.014*
Who would you call first if you ran out of medication and there were no refills left on your bottle? (“doctor or pharmacy” was considered independent action)	Age (years)	17.68 ± 1.76	16.55 ± 1.60	0.01
	Parents older than 40 years			
	Yes	70.40 %	92.50 %	0.02
	Driver’s license			
	Yes	64.50 %	29.30 %	0.03*
Who makes your routine doctor’s appointments? (“yourself” was considered independent action)	Age (years)	18.5 ± 1.2	16.5 ± 1.7	<0.0001
	Fibromyalgia			
	Yes	61.50 %	11.70 %	0.02*
	Non-rheumatologic diagnosis			
	Yes	75 %	33.30 %	0.01
What do you do if you can’t keep an appointment scheduled with your rheumatologist? (“call and cancel yourself” was the independent action)	Age (years)	18.51 ± 1.23	16.3 7 ± 1.58	<0.0001
	3Disease duration (years)	5.77 ± 3.66	3.91 ± 2.97	0.03
	Family with similar disease			
	Yes	72.10 %	44.70 %	0.03
	Driver’s license			
	Yes	77.30 %	37.50 %	0.003*
	Summer job			
	Yes	90.90 %	43.10 %	0
Do you take your medications as prescribed without someone reminding you?	No significant variable associations			

*No longer significant when controlled for age

**Table 3:** Variables associated with greater self-reported independence in accessing health system in pediatric rheumatology patients

Older age	
Longer disease duration	
Family member with similar condition	
Younger age of parent	
Ever had a summer job	
Co-morbid non-rheumatic diagnosis	

Respondents who stated that they would call the doctor if they were not feeling well were older than those who stated they would not (18.8 versus 16.7 years of age; *p* = 0.002). Having had a summer job was initially significant for this independent behavior (100 % versus only 51.5 % of those who did not respond independently), but when controlled for age, it was no longer significant. Young adults who reported they would call the doctor or pharmacy if refills were required were older (17.7 versus 16.6 years of age; *p* = 0.005). Interestingly, these same patients were less likely to be independent in this measure if their parents were older than 40 years of age (70.4 % vs. 92.5 %; *p* = 0.02). Those with driver’s licenses were more independent in this measure (64.5 % vs. 29.3 %; *p* = 0.03), but this variable was no longer significant when controlled for age.

Those patients who reported they would schedule their own physician visits were older (18.5 vs. 16.5 years; p < 0.0001) and more often had a non-rheumatologic diagnosis (75 % vs. 33.3 %; *p* = 0.008). Variables significant for independence in calling to cancel the doctor’s visit if unable to attend included older age (18.5 vs. 16.3 years; p < 0.0001), longer disease duration (5.8 vs. 3.9 years; *p* = 0.03), having a family member with a similar disease (72.1 % vs. 44.7 %; *p* = 0.03), and having had summer job (90.0 % vs. 43.1 %; *p* = 0.0002). Having a driver’s license was also initially significant (77.3 % vs. 37.5 %; *p* = 0.003), but was not when controlled for age.

Three specific rheumatologic diagnoses (JIA, lupus, and fibromyalgia) were analyzed to see if a particular diagnosis resulted in greater or less likelihood of being independent in obtaining medical care. The only diagnosis that was significantly associated with any independence outcomes was fibromyalgia. Patients with fibromyalgia were more likely to make their own doctor appointments than those without fibromyalgia (61.5 % versus 11.7 %; *p* = 0.02); however, when controlled for age, this was no longer significant. The mean age of patients with fibromyalgia was 17.6 years vs. 16.8 years for patients without fibromyalgia (*p* = NS).

Screening for additional associations, we collected pain scores and patient global assessment (PGA) of overall well-being scores. We then performed stepwise regression analysis to see if the answers to the following questions predicted a higher or lower pain or PGA score: “Does your mother or father give you your medications to take?”, “Who do you tell or call first if you feel your disease is active or worsening?”, “Who would you call first if you ran out of medication and there were no refills left on your bottle?”, and “What do you do if you can’t keep an appointment scheduled with your rheumatologist?”. None of the *p*-values for these comparisons were significant. Therefore, in our study, pain and PGA scores were not associated with more or less independence in obtaining medical care.

## Discussion

Since many adolescents with a rheumatic diagnosis have active disease into adulthood, effective transition is essential. One study of patients with JIA, the most common childhood rheumatic disease, aged 17 years of age, found that 80 % still had active disease [15]. Hence, smooth transition of patients from pediatric to adult providers is critical to ensure continuation of care and improved outcomes. Knowing when to transfer a patient, particularly one who might have persistent disease activity, is challenging.

In addition to our data, other published studies have looked at transition readiness in pediatric patients with chronic disease. In California, one center analyzed readiness for transition in 52 adolescents in a pediatric rheumatology clinic [16]. They looked at independence in self-management tasks and self-reported medication adherence in 13–16 year olds versus 17–20 year olds. This study noted an age related increase in certain skills such as ability to fill prescriptions, schedule appointments, arrange transportation, ask questions of doctors, manage medical insurance, and recognize symptoms of illness. However, other skills, such as taking medications as prescribed, keeping a calendar of appointments, and keeping a personal health file did not improve with age. A survey of patients with juvenile myositis showed that patients over 15 years of age were more likely to make own doctor appointments, take medications without being reminded, know when to seek medical attention, and know how to refill medications [17].

Our study demonstrates that older age correlates with self-reported independence in almost all of the questions asked. Older patients were more likely to report they would schedule their own doctor appointments, call the doctor himself/herself if not feeling well, call to cancel appointments if could not attend, and call the doctor or pharmacy if refills were required. A strength of our study included our sample’s age composition, as we had a wide range of ages and a significant number of older adolescents; in fact, at least half the patients were 18 years of age or older. Our study population may be slightly older than other pediatric practice based studies, demonstrating that perhaps some of these skills are attained at a later age. In one study, evaluating 16–26 year olds with special health care needs, older age was associated with higher scores in self-management [18]. Since our patients were being seen in a pediatric clinic, there may be some sample bias in that these patients had not transferred to adult care despite being up to age 21. At our institution, there is not a standard transition process, so different providers may transition patients at different ages depending on many variables such as patient’s disease activity, patient/parent proactivity in seeking adult provider, and patient/provider comfort level.

One weakness of this study is that some of the groups analyzed had small numbers of patients such as patients with parents <40, patients who call the doctor himself/herself when not feeling well, or patients who need reminder to take medications. We did not have enough patients living independently to assess how this affected independence in obtaining healthcare. All patients but one still lived at home, despite 18 % being in college. Our patients may represent a sample that is delayed in transition readiness already compared to the norm. Our population was primarily Caucasian, so our data may not be applicable to other racial groups, as there may be differences in transition readiness.

Our questionnaire has some limitations in that it was created by surveying pediatric and adult rheumatologists at our institution on which variables they felt resulted in successful transition. We are a unique practice in that many youth transition from pediatric to adult rheumatology while staying at our institution, so we are well poised to observe and discuss the transition difficulties of our patients. Nevertheless, the determination of questionnaire items may have been biased by the specific opinions and experience of these seven providers. Moreover, nurses and other allied health providers, who may play a significant role in educating patients and assisting with transition, were not included in this exercise. In our practice, nurses and other allied health providers do not participate in many of the clinic visits, but certainly the input of all health care providers as well as patients and parents would be preferred in the creation of a questionnaire. Although not available at the time of this study, there are now available published validated transition questionnaires. The transition readiness assessment questionnaire (TRAQ) is an example of a published transition readiness tool used in youth with special healthcare needs [18]. This tool has 20 questions that assess skills for self-management and self-advocacy. Our questionnaire is unique in that in addition to asking many questions related to independence in managing medical care, our survey includes more demographic, life skills, and disease feature items than the TRAQ, thus allowing us to look at additional patient variables that may impact transition readiness. We were able to analyze how these different demographic, disease feature, and life skill characteristics correlated with self-reported independence in managing one’s own health care.

In addition to older age, we found other variables that correlated with independence in obtaining medical care, including having a co-morbid non-rheumatology diagnosis, longer disease duration, younger parents, and family member affected with a medical condition. Fifty-four percent of our patients reported that they had a family member with a “similar disease”, and this correlated with self-reported independence in obtaining medical care. We surveyed our patients about family members having a “similar illness” as we wondered if these patients might be more experienced or used to dealing with the discipline of rheumatology and the medical field in general and thus might be more willing to direct their own care. However, we did not define what we meant by “family member” or “similar disease,” so this was left to patient interpretation. Young adults who are accustomed to being surrounded by a medical environment due to having a sick family member (regardless of the nature of the medical issue) could potentially be more proactive and comfortable accessing medical care. Our study also found that when the youth reported on the questionnaire that they had a comorbid non-rheumatologic diagnosis, this correlated with them answering that they scheduled their own routine doctor appointments. We did not collect information on what non-rheumatology medical diagnoses the patients had, nor did we verify that they had a non-rheumatology diagnosis. Nevertheless, if a young adult has had many medical encounters due to several different diagnoses or symptoms, he/she may be more independent in obtaining medical care. Our study found that having had a longer disease duration correlated with self-perceived independence in obtaining medical care. By the same reasoning mentioned above, if a teenager has had a longer disease duration and hence more contact with the medical system, then he/she may be more used to how things work and may self-navigate in that system. Parents may feel more comfortable and allow youth to operate independently if they have had time to get used to the diagnosis and have seen their child do okay over time.

We found that teenagers/young adults with a parent younger than the age of 40 reported more independence in accessing medical care. One possible explanation is that younger parents might be less overprotective than older parents in caring for their children with chronic health care needs. Young parents may be more likely to have young children, such as infants, who demand their time, thus leaving the parent less time to micro-manage the affairs of their teenager with a chronic medical condition. Younger parents may have less financial stability and may work more jobs or less desirable hours than older parents, and thus may allow/encourage their teenagers to be more independent. In our study, when the patients had had a summer job, they were more likely to be independent in obtaining health care. It makes sense that teenagers who have worked may feel more comfortable making phone calls to an organization and interacting with adults without parents present. They may be more accustomed to keeping a schedule of appointments.

Our independence-related questions may be biased by the patients’ opinions of their own abilities, behaviors, and/or knowledge. They may over or underestimate their willingness and tendency to perform independently. We recognize that our independence questions only assess self-reported independence in accessing the health system. Further study is needed to determine if these variables actually correlate with increased autonomy and successful transition. A similar study demonstrated that adolescents’ attitude toward transition and their level of self-efficacy in managing day-to-day care and hospital visits were strongly associated with self-reported transition readiness [19].

Management of health and disease is only one aspect of successful transition to adulthood. Young adults with chronic medical conditions are at risk for poor educational, vocational, and financial outcomes [11]. Transition planning needs to consider other aspects such as job training, securing of health insurance, and independent living skills. Young adults perceive transition needs to be greater than just physical health, as they want social, psychological, and vocational issues considered [20]. Our study did not address issues besides management of disease and medical care.

A recent clinical report provides expert consensus on how to implement transition and a comprehensive algorithm for managing the transition process [21]. The idea of transition should be introduced to patients by age 12–13 and discussed regularly, as transition should be a gradual process. In addition to developing an individualized transition plan for each patient that is reviewed and updated over time, essential components include having a transition policy that is visible to patients, maintaining an up-to-date portable medical summary, identifying personnel in the office responsible for transition tasks/care coordination, use of a transition readiness assessment tool, use of a transfer checklist, and documentation of the transfer [21]. All of these components are important when seeking to improve the transition process, and a comprehensive approach is more likely to result in successful transition. There has been at least one organized quality improvement initiative that evaluated pediatric and adult primary care practices on performance on 6 accepted quality indicators for transition, using the “Six Core Elements of Health Care Transition” [7]. Transition should become a top quality improvement focus as we work toward optimizing care and improving outcomes for patients with chronic disease

## Conclusion

Our study found that older age, having a parent less than age 40, having a family member with a similar disease, a longer disease duration, having had a summer job, and having a comorbid non-rheumatic diagnosis correlated with increased self-reported independence in obtaining medical care in pediatric rheumatology patients. Our study was unique in that it assessed a wide range of demographic, disease-related, and life skill characteristics that could affect a patient’s tendency to be independent in managing one’s own medical care. Healthcare providers should consider the variables we found significant when assessing patients for transition readiness.

## Abbreviations

JIA: Juvenile Idiopathic Arthritis
AAP: American Academy of Pediatrics
PGA: Patient Global Assessment
TRAQ: Transition Readiness Assessment Questionnaire
